# Case report: Bridging radiation therapy before chimeric antigen receptor T-cell therapy induces sustained remission in patients with relapsed/refractory double-expressor diffuse large B-cell lymphoma with localized compressive symptoms

**DOI:** 10.3389/fimmu.2024.1441404

**Published:** 2024-09-03

**Authors:** Liying Yang, Mengdi Wu, Hao Yang, Xiaorong Sun, Lijie Xing, Dan Liu, Ligang Xing, Jinming Yu

**Affiliations:** ^1^ Shandong University Cancer Center, Shandong University, Jinan, Shandong, China; ^2^ Department of Radiation Oncology, Shandong Cancer Hospital and Institute, Shandong First Medical University, and Shandong Academy of Medical Sciences, Jinan, Shandong, China; ^3^ Department of Graduate, Shandong First Medical University and Shandong Academy of Medical Sciences, Jinan, Shandong, China; ^4^ Department of Nuclear Medicine, Shandong Cancer Hospital and Institute, Shandong First Medical University and Shandong Academy of Medical Sciences, Jinan, Shandong, China; ^5^ Department of Hematology, Shandong Cancer Hospital and Institute, Shandong First Medical University and Shandong Academy of Medical Sciences, Jinan, Shandong, China

**Keywords:** lymphoma, double-expressor diffuse large B-cell lymphoma, chimeric antigen receptor T-cell immunotherapy, radiotherapy, bridging therapy

## Abstract

**Background:**

High-risk double-expressor diffuse large B-cell lymphoma has an inferior prognosis following standard first-line therapy. After failure of second-line therapy, treatment options are limited if accompanied by localized compressive symptoms. Chimeric Antigen Receptor T cell (CAR-T) therapy preceded by bridging radiotherapy may be an effective emerging therapy.

**Case presentation:**

We report a 66-year-old female patient diagnosed with stage IV double-expressor diffuse large B-cell lymphoma. The patient achieved progressive disease after two cycles of rituximab, cyclophosphamide, liposomal doxorubicin, vincristine, and prednisone and continued to develop cervical lymph node recurrence after second-line therapy. The patient was infused with CAR-T cells after receiving focal bridging radiotherapy and remained in complete response more than 9 months after treatment. In addition, the patients did not experience serious adverse reactions related to radiotherapy as well as CAR-T cell therapy.

**Conclusions:**

In this article, we describe a patient with double-expressor diffuse large B-cell lymphoma with localized compression symptoms after second-line treatment failure who benefited from CAR-T combined with focal bridging radiotherapy.

## 1 Introduction

Diffuse large B-cell lymphoma is an aggressive tumor derived from mature B cells, accounting for approximately 30% to 40% of all non-Hodgkin lymphomas (1). After treatment with first-line rituximab, cyclophosphamide, doxorubicin, vincristine, and prednisone regimen, 45% to 50% of patients will experience relapse or refractory treatment (2, 3). Asthe number of treatment lines increase, about 73% of patients with relapsed/refractory diffuse large B-cell lymphoma still cannot obtain remission after receiving second-line and later-line treatments (4–6). Among them, double-expressor diffuse large B-cell lymphoma, as a high-grade lymphoma, is more aggressive and has a worse prognosis (7). A consensus on double-expressor diffuse large B-cell lymphoma treatment is still lacking. Chimeric Antigen Receptor T cell (CAR-T) targeting CD19 has been approved by the United States Food and Drug Administration for the treatment of relapsed/refractory diffuse large B-cell lymphoma, with a 1-year recurrence-free survival of approximately 44% to 65% (8–10). Bridging radiotherapy is expected to further improve the recurrence-free survival (11), which double-expressor diffuse large B-cell lymphoma patients bring a therapeutic ray of hope. Here, we report a 68-year-old woman with dual-expression type relapsed refractory lymphoma who successfully transitioned to CAR-T cell therapy with local bridging radiotherapy and has achieved a 9-month complete remission to date. To our knowledge, this is the first report of CAR-T cell infusion using localized radiotherapy as a transitional therapy for the treatment of relapsed/refractory dual-expression lymphoma.

## 2 Case presentation

**Figure 1:**
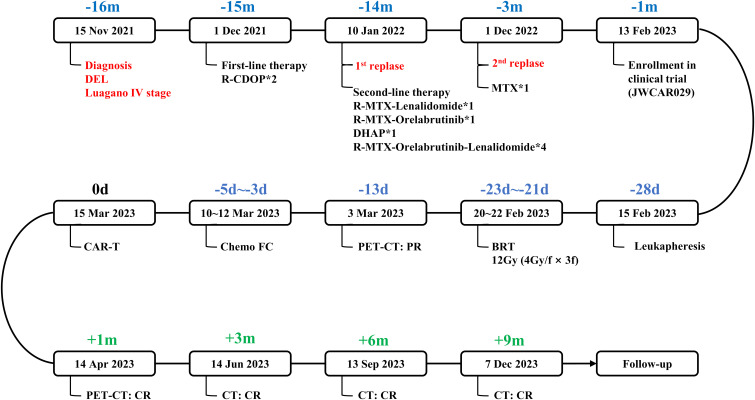
Flow chart of the disease process and therapeutic modalities. DEL, double-expressor diffuse large B-cell lymphoma. R-CDOP, rituximab, cyclophosphamide, liposomal doxorubicin, vincristine, and prednisone. methotrexate, methotrexate. BRT, bridging radiotherapy. FC, fludarabine and cyclophosphamide. PR, partial response. CR, complete response. R-CDOP*2, two cycles of R-CDOP treatment. R-MTX-Lenalidomide*1, one cycle of R-MTX-Lenalidomide treatment. R-MTX-Orelabrutinib*1, one cycle of R-MTX-Orelabrutinib treatment. DHAP*1, one cycle of DHAP treatment. R-MTX-Orelabrutinib-Lenalidomide*4, four cycle of R-MTX-Orelabrutinib-Lenalidomide treatment. MTX*1, one cycle of MTX treatment.

The patient received a diagnosis of double-expressor diffuse large B-cell lymphoma in November 2021, at the age of 66 years, after presenting with fatigue. She received chemoimmunotherapy with 2 cycles of rituximab, cyclophosphamide, liposomal doxorubicin, vincristine, and prednisone which resulted in progressive disease in the central nervous system (as reported by the patient, no report available). Subsequent treatment included regimens incorporating rituximab, methotrexate, lenalidomide, and orelabrutinib (**Figure 1**). On February 1, 2023, a biopsy of the left cervical mass diagnosed high-grade B-cell lymphoma. Immunohistochemical staining showed: CD3 (-), CD5 (+), CD20 (+), PAX5 (+), CD10 (-), BCL-2 (+), BCL-6 (50%+) MUM-1 (-), C-MYC (40%+), CyclinD1(-), Ki-67 (90%+), EBER (-). Upon admission to our hospital, the International Prognostic Index (IPI) score was 3.

**Figure 2:**
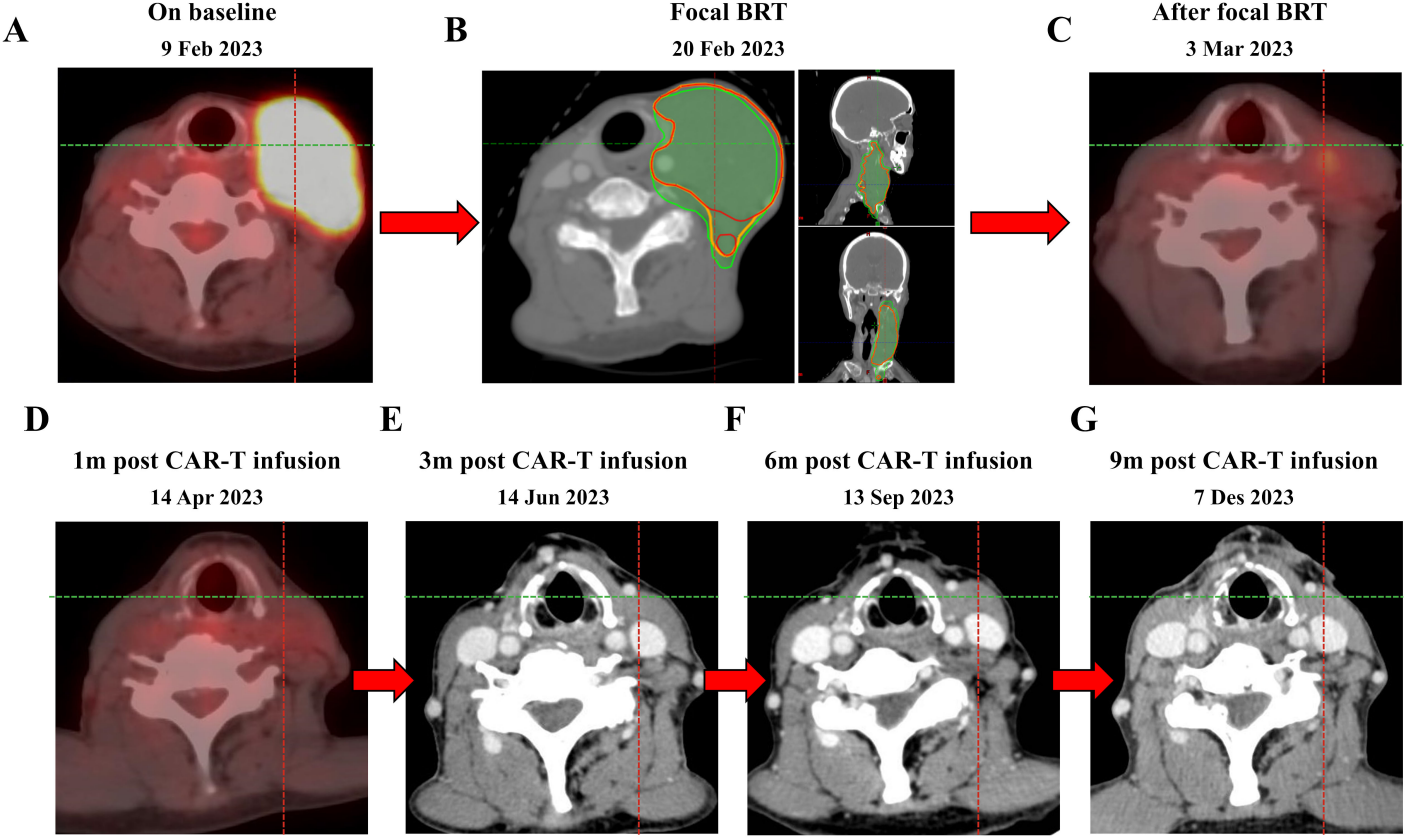
PET-CT scan before bridging radiotherapy (9 Feb 2023) **(A)**, bridging radiotherapy treatment (20 Feb 2023) **(B)** and after bridging radiotherapy treatment (3 Mar 2023) **(C)**. The patient remains in CR with CAR-T therapy to date **(D–G)**. bridging radiotherapy, bridging radiotherapy. GTV, gross tumor volume. CTV, clinical tumor volume. PTV, planning target volume. The red line represents GTV, the yellow line represents CTV, and the green line represents PTV.

Approximately 11 months later, the disease progressed again and a physical examination revealed a mass about 5cm in diameter in the neck. ^18^F-Fluorodeoxyglucose positron emission tomography/computed tomography showed multiple enlarged lymph nodes in the left neck and left supraclavicular region with a maximum diameter of 4.7 cm and a standard uptake volume of 24.0 (**Figure 2A**). Give methotrexate treatment for 1 cycle. Considering the reported anti-lmphoma effects of anti-CD19 CAR-T cells and the failure of second-line therapy in this patient, we encouraged her to enroll in a clinical trial of anti-CD19 CAR-T treatment to which she agreed (Ethics NO. SDZLEC2022-164-01). At the same time, the multidisciplinary team recommended local bridging radiotherapy to relieve the compression symptoms of neck mass, and the patient agreed.

A large-aperture CT was performed for supine positioning, a large mask was immobilized, and a laser light system was used to identify body surface marker points. The left supraclavicular and left cervical lymph nodes were outlined as GTV, with 3 mm of externals as CTV and 5 mm of externals as PTV. The prescribed dose of 400 cGy/dose × 3 sessions was drawn up and the TPS developed an IMRT plan. Evaluating the radiotherapy plan at a total of 1,200 cGy, the isodose curve of 1,200 cGy encompassed 95% of the PTV, with a mean dose to the right parotid gland of 103.8 cGy, a mean dose to the left parotid gland of 627.7 cGy, and a maximal point dose to the spinal cord of 533.8 cGy, with the remaining critical organs in the tolerable range. There was no discomfort during radiotherapy, and the cervical lymph nodes on examination after radiotherapy were significantly smaller and softer than before (**Figure 2B**). Post-radiotherapy PET-CT showed (March 3, 2023) left supraclavicular and left cervical lymph node involvement was reduced after treatment compared to before (February 9, 2023), metabolism was reduced (**Figure 2C**).

Chemotherapy (fludarabine 36mg qd and cyclophosphamide 360mg qd) was administered 5, 4, and 3 days before the first infusion of CAR-T cells (March 15, 2023) which was followed by one infusion administered. The effective anti-CD19 CAR-T cells totaled 100 * 10^6^. The patient developed fever once on March 20, 2023, with a maximum body temperature of 38.0°C, which dropped to normal on its own. A grade 1 cytokine release syndrome reaction occurred on March 24, 2023, and granulocyte colony stimulating factor was given to increase white blood cells. Furthermore, the patient did not develop grade 3-4 cytokine release syndrome or immune effector cell-associated neurotoxic syndrome. The patient remained complete response for more than 9 months after anti-CD19 CAR-T reinfusion (**Figures 2D–G**).

## 3 Discussion and conclusions

Our work demonstrated that receiving anti-CD19 CAR-T cell infusion after focal bridging radiotherapy had a significant effect in patients with double-expressor diffuse large B-cell lymphoma. The double-expressor diffuse large B-cell lymphoma case we described had a rapidly that showed poor response to conventional therapy. She responded favorably to anti-CD19 CAR-T cells therapy. To the best of our knowledge, this is the first case report of second-line relapsed double-expressor diffuse large B-cell lymphoma treatment with anti-CD19 CAR-T cell with bridging radiotherapy. Complete response was still observed 9 months after CAR-T infusion, and no treatment is expected to prolong complete response duration. In addition, the case did not experience serious adverse events.

Refractory/relapsed lymphomas are highly heterogeneous and require constant experimentation with new modes of diagnosis and evaluation. CAR-T cell therapy is the treatment of choice for patients with refractory relapsed, post-transplant relapsed, and salvage treatment failures/relapses, with the number of lines of therapy constantly moving forward. Currently, in the field of double-expressor diffuse large B-cell lymphoma, CAR-T cells have demonstrated favorable efficacy in treating high-risk diffuse large B-cell lymphoma patients (12–14). The ZUMA-12 study included 40 patients with high-risk diffuse large B-cell lymphoma treated with CD19 CAR-T cells and showed an objective response rate of 89% and a complete response rate of 78% in 37 evaluable patients (15). Still about 30% fail to obtain a CR before waiting for the CAR-T cells to be manufactured. Therefore, the “bridging therapy” from cell harvesting to clearing chemotherapy and successful CAR-T infusion may be a key factor in controlling the progression of the disease and influencing the clinical outcome (11).

Multiple retrospective studies have found that in patients with diffuse large B-cell lymphoma, overall survival is significantly prolonged in patients who receive bridging therapy compared to those who do not (16–18). Comparison of different bridging modalities revealed that bridging radiotherapy was superior to other bridging treatment modalities (17–21), which may be related to the unique biological effects of radiotherapy (11). Radiotherapy can directly inhibit the ability of tumor cells to divide and proliferate by damaging their DNA, while also promoting the migration and activation of pro-inflammatory immune cells toward irradiated areas of the tumor (22–24). The above retrospective clinical studies confirm that CAR-T combined with bridging radiotherapy may be a safer and more effective treatment modality for diffuse large B-cell lymphoma, but the optimal timing, site, dose, and modality of radiotherapy still need to be explored (25–27).

Currently, many international clinical trials of CAR-T combined with bridging radiotherapy have been conducted, and these clinical trials are mainly in phase I (28–34), with the timing of radiotherapy focusing on the period between single-treatment and clear lymphoma (28, 29, 32–35), and there are also a few salvage therapies after the failure of CAR-T treatment (36). Comprehensive radiotherapy, i.e., radiotherapy to all high uptake sites shown by PET-CT, is the mainstay of radiotherapy, and there are also clinical trials related to Focal radiotherapy. Radiotherapy doses were based on conventional fractionated radiotherapy (28, 31, 32, 35) and hypofractionated radiotherapy (28, 29, 33). Compared with conventional fractionated radiotherapy, hypofractionated radiotherapy can better mobilize local and systemic immune responses and remodel the tumor microenvironment by inducing immune cell infiltration (37). Local radiotherapy also removes tumor-resident lymphocytes, providing space for CAR-T cells to infiltrate (11, 38). There are no investigational trials demonstrating the safety and efficacy of CAR-T in combination with hypofractionated radiotherapy-bridged radiotherapy in patients with double-expressor diffuse large B-cell lymphoma who have failed second-line therapy with large localized masses, and new clinical trials are needed to investigate the safety and efficacy of this treatment option.

Although our case found that bridging radiotherapy before CAR-T infusion may be an effective treatment for patients with localized compressive symptoms, in actual clinical practice, the choice between bridging radiotherapy and early CAR-T cell therapy needs to balance multiple factors. Radiotherapy can control locally progressive disease, alleviate symptomatic disease, and clear high metabolic tumors without increasing the toxicity of subsequent CAR-T infusion (39). However, early CAR-T cell therapy is also a potential option, especially when patients respond poorly to conventional treatment regimens and the disease progresses rapidly. Direct CAR-T cell therapy can reduce treatment delays, rapidly intervene to control tumor burden, and potentially avoid acute and chronic toxicities associated with radiotherapy. Therefore, an individualized bridging strategy should be developed based on the patient’s condition (whether the disease is stable, whether there are large masses or symptomatic lesions).

In conclusion, bridging hypofractionated radiotherapy before CAR-T treatment may be a safe and effective treatment option for patients with double-expressor diffuse large B-cell lymphoma who have failed second-line therapy and have localized compression symptoms.

## Data Availability

The original contributions presented in the study are included in the article/supplementary material. Further inquiries can be directed to the corresponding author.
